# Integrating High-Value Care and Environmental Sustainability to Reduce Unnecessary Laboratory Testing by Residents: An Interventional Pilot

**DOI:** 10.1007/s11606-025-09776-0

**Published:** 2025-08-04

**Authors:** Peter Trinh, Joshua E. Lewis, Julie Fiskio, Jeffrey L. Schnipper, Paul F. Dellaripa, Herrick Fisher

**Affiliations:** 1 Department of Medicine, Brigham and Women’s Hospital, Boston MA, USA; 2 Department of Pathology, Brigham and Women’s Hospital, Boston, MA USA; 3 Hospital Medicine Unit, Division of General Internal Medicine and Primary Care, Brigham and Women’s Hospital, Boston, MA USA; 4 Harvard Medical School, Boston, MA USA; 5 Division of Rheumatology, Department of Medicine, Brigham and Women’s Hospital, Boston, MA USA

## Abstract

**Background:**

Routine lab testing of stable inpatients represents low-value care and contributes toward healthcare’s significant carbon footprint. If the US healthcare system was its own nation, it would be the 13th largest emitter of greenhouse gases. Integrating the concepts of high-value care and environmental sustainability represents a potentially new approach to reduce unnecessary lab testing, low-value care, and carbon emissions.

**Objective:**

To assess if an intervention integrating concepts of environmental sustainability and high-value care can influence resident lab ordering practices.

**Design:**

We conducted a 6-month concurrent control versus intervention pilot. Statistical analysis consisted of matched-pair propensity score analysis and Wilcoxon rank-sum test.

**Participants:**

Two medicine teams with equal composition of attending and resident physicians at an urban academic tertiary care center.

**Interventions:**

Intervention team received (1) a video on the negative effects of unnecessary testing, including adverse environmental impacts; (2) daily team-based reviews of lab orders; and (3) reminder emails about team-based reviews. Intervention team residents received quantitative surveys to assess ordering behaviors.

**Main Measures:**

Primary outcomes were the average number of both basic lab tests and venipunctures per patient per day. Secondary outcomes included kilograms of carbon dioxide (CO_2_) saved and resident perceptions of ordering practices.

**Key Results:**

Control and intervention patients, respectively, had on average 3.91 versus 3.62 labs per patient day (*p* < 0.001) and 1.39 versus 1.32 venipunctures per patient day (*p* = 0.003). An estimated 81 kg CO_2_ was saved. The proportion of surveyed residents who assessed clinical lab indications at least multiple times weekly or daily increased from 47.8% pre-intervention to 86.9% post-intervention (*p* = 0.03).

**Conclusion:**

An intervention integrating high-value care and environmental sustainability changed resident ordering behavior, producing fewer lab tests and venipunctures and a smaller carbon footprint. This approach represents an additional modality to reduce unnecessary laboratory testing and low-value care.

**Graphical Abstract:**

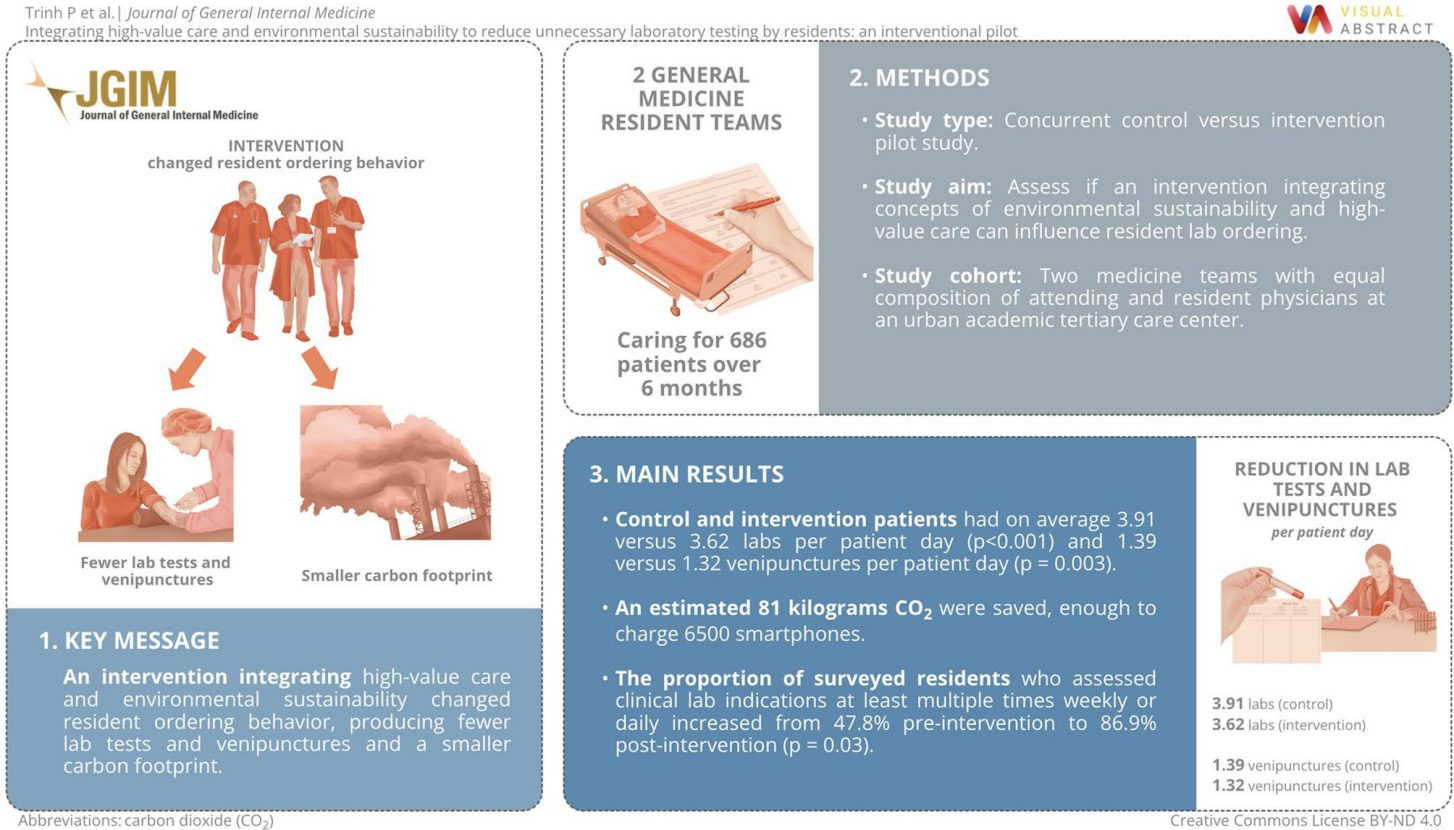

**Supplementary Information:**

The online version contains supplementary material available at 10.1007/s11606-025-09776-0.

## INTRODUCTION

The American Board of Internal Medicine and the Society of Hospital Medicine have identified routine lab testing of stable inpatients as a source of low-value care.^1,2^ As it is estimated that upwards of 21% of all lab tests represent inappropriate overutilization,^3^ reducing unnecessary testing could have a significant impact on cost saving, limiting waste, and improving healthcare quality. Beyond minimizing iatrogenic anemia and reducing costs,^4,5^ another benefit of reducing superfluous lab testing is decreasing healthcare’s carbon footprint. If the US healthcare system were its own nation, it would be the 13th largest emitter of greenhouse gases.^6^ A basic metabolic panel is associated with approximately 99 g of carbon dioxide (CO_2_) emissions,^7^ which are derived from the production, transportation, and disposal of lab materials as well as the processing of the lab test itself. Given the volume of inappropriate tests performed annually, reducing unnecessary lab testing at scale could have a meaningful impact on the carbon footprint of US healthcare. Prior resident-led projects have emphasized education on clinical indications, patient experience, and “mindful ordering” practices to reduce unnecessary lab ordering.^8–11^ The following prospective cohort pilot was designed to assess whether a multifaceted behavioral and educational intervention integrating the concepts of environmental sustainability and high-value care could influence resident lab ordering practices for clinically stable inpatients.

## METHODS

### Data and Study Population

We conducted a 6-month intervention pilot on two general medicine resident teams at a tertiary care academic medical center in Boston, MA. Residents spent 1 month total on the respective study teams. Over the 6-month study period, there was no crossover of residents between the two teams, but there was crossover of attendings, and one resident participated in the intervention team twice. Basic lab tests refer to the basic metabolic panel (BMP), complete blood count (CBC), magnesium, phosphorus, liver function tests (LFTs), and prothrombin time international normalized ratio (PT-INR). Phlebotomy and lab data were extracted from the electronic medical record (Epic Corporation). Data were excluded if labs were ordered by non-medicine team members, if lab draws were conducted for arterial blood gases or fingerstick glucose checks, if patient length of stay (LOS) was less than 24 h at the time of lab draw, or if lab draws were conducted within 12 h of an intensive care unit (ICU) transfer, rapid response event, code, or operating room visit. Per the Mass General Brigham (MGB) institutional review board (IRB), this study was exempt from IRB approval as it did not meet human subject research criteria and is not intended for generalized knowledge.

### Procedure

One resident team served as the control, and the other served as the intervention arm. Each team had the same composition of attending and resident physicians and covered 16 inpatient beds. The intervention residents (1) viewed an educational video on the effects of unnecessary lab testing with a focus on the adverse environmental impacts of US healthcare at the beginning of their monthlong rotation, (2) implemented daily team-based reviews of basic lab orders, and (3) received thrice weekly emails as reminders to perform the team-based reviews. Residents on the intervention team received anonymous pre- and post-implementation surveys to assess attitudes and ordering behaviors. Pre- and post-surveys were administered at the start of the rotation and the end of the 1-month rotation, respectively. The pre- and post-intervention surveys were sent to the resident who served twice on the intervention team only during their first time on the intervention team. The 14-question multiple-choice survey was designed by the project team (Supplement 1) and modeled on work by Sedrak et al.^12^ The surveys assessed perceived institutional culture regarding lab ordering, personal lab ordering practices, and barriers to decreasing unnecessary labs. The post-implementation survey also included a question in which residents ranked how motivating six factors were to their lab ordering practices via a 6-point Likert scale. Survey data were collected and managed using REDCap electronic data capture tools hosted at MGB.^13,14^

### Outcomes

The primary outcomes were the average number of basic lab tests per patient per day and average venipunctures per patient per day. The secondary outcomes were estimated kilograms of CO_2_ saved and resident perceptions of lab ordering practices.

### Statistical Analysis

The number of basic labs and venipunctures per day was calculated for each patient. These values were averaged across all patients in the control and intervention cohorts. To assess for statistically significant differences, a matched-pair propensity score analysis was conducted, adjusting for demographics (age, sex, ethnicity, race, language) and clinical covariates of number of comorbidities and Elixhauser comorbidity index (Van Walraven modification)^15^. Propensity scores were calculated via a logistic regression model predicting intervention group based on covariate values. Matched pairs between individuals within the control and intervention groups were made based on propensity score similarity using nearest neighbor without replacement. A two-tailed paired *t*-test with pairs comprised of propensity score-matched pairs was performed for each outcome of interest. For the survey data, a Wilcoxon rank-sum test was performed to determine statistical significance. A two-sided *p*-value of < 0.05 was used as the threshold for statistical significance. Python software was used for all analyses.

### Carbon Footprint Analysis

Carbon footprint was estimated using data from McAlister et al.^7^ and the US Environmental Protection Agency’s Greenhouse Gas Equivalencies Calculator.^16^

## RESULTS

During the 6-month study period, the two teams cared for 686 total patients: 333 and 353 patients by the control and intervention teams, respectively (Table 1). The control patient cohort was 2.8 years older on average (*p* = 0.044). Other baseline patient demographics were similar between groups. There was no statistical difference in medical complexity between the two patient cohorts as determined by Elixhauser/Van Walraven score.

**Table 1:** Baseline Patient Characteristics

Category	Control	Intervention	*p*-value
Patients	333	353	–
Patient days	2363	2129	–
Age in years (IQR)	64.0 (50–78)	61.2 (47–76)	0.044
Male sex (%)	156 (47)	162 (46)	1.000
Non-Hispanic ethnicity — no (%)	279 (84)	314 (89)	0.143
Race — no. (%)			0.779
White	53 (16)	45 (13)	
Black	63 (19)	74 (21)	
Asian	13 (4)	14 (4)	
Unknown	206 (62)	218 (62)	
English speaking (%)	85%	89%	0.139
Comorbidities (IQR)	5.0 (3–7)	4.8 (3–6)	0.390
Val Walraven score (IQR)	12.1 (4–19)	12.2 (5–19)	0.891

### Lab Test Outcomes

Control team patients had on average 3.91 labs per patient day compared to 3.62 labs per patient day for intervention team patients, a 7.4% difference (*p* < 0.001) (Table 2). Control patients had on average 1.39 venipunctures per day compared to 1.32 venipunctures per day for intervention patients, representing a 5% difference (*p* = 0.003). This amounted to an estimated 81-kg (kg) reduction in CO_2_ emissions over 6 months.^7^

**Table 2:** Average Number of Labs and Venipunctures Per Patient Per Day

	Control (95% CI)	Intervention (95% CI)	***p***-value
Number of patients	333	353	–
BMP	1.15 (1.12–1.18)	1.11 (1.08–1.14)	0.072
Magnesium	0.94 (0.92–0.97)	0.88 (0.85–0.90)	< 0.001
Phosphorus	0.43 (0.41–0.45)	0.45 (0.43–0.48)	0.082
LFTs	0.56 (0.54–0.58)	0.47 (0.45–0.49)	< 0.001
CBC	0.47 (0.44–0.49)	0.38 (0.36–0.41)	< 0.001
PT-INR	0.36 (0.34–0.38)	0.33 (0.30–0.35)	0.003
Total labs	3.91 (3.82–4.00)	3.62 (3.52–3.71)	< 0.001
Total venipunctures	1.39 (1.36–1.43)	1.32 (1.28–1.36)	0.003

### Resident Surveys

Twenty-three out of 35 residents on the intervention team responded to both pre- and post-implementation surveys (66% response rate). Results showed some improvements in attitudes toward high-value care and decreased ordering of unnecessary lab tests amongst these residents (Supplement 2 Table 1). Pre-intervention, 73.9% of surveyed residents agreed or strongly agreed that they maintained clinically unnecessary lab orders on their patients. This decreased to 39.1% post-intervention (*p* = 0.03). There were also increases in the percentage of residents who self-reported conducting the following discrete actions multiple times per week or daily: reviewing and assessing clinical indications for basic lab tests (47.8% pre-intervention to 86.9% post-intervention, *p* = 0.03), decreasing frequency of basic lab tests (60.8% pre-intervention to 82.6% post-intervention, *p* = 0.15), and fully discontinuing basic lab tests for clinically stable patients (43.4% pre-intervention to 60.9% post-intervention, *p* = 0.25). The percentage of residents who agreed or strongly agreed that the lab ordering culture of the residency program embodied high-value care also increased from 47.8% pre-intervention to 65.2% post-intervention, but this difference was not statistically significant (*p* = 0.27).

Regarding which factors were the most motivating for residents to engage in lab reviews and lab order de-escalation, respondents on average ranked environmental sustainability fourth behind lack of clinical indications, patient experience, and patient safety, respectively, and ahead of financial savings and perceived expectations of team members (Table 3). Respondents also identified barriers that contributed to any reluctance to de-escalate lab orders. In the post-survey, the top five factors were not wanting to miss changes in patient clinical status, discomfort with diagnostic uncertainty, insufficient time to review patient lab orders, lack of cost transparency of labs, and lack of cost-conscious culture at the institution (Supplement 2 Table 2).

**Table 3:** Factors Motivating Intervention Residents to De-escalate Labs

Motivating factor	Average Likert score*
Lack of clinical indications	2.26
Patient experience	2.61
Patient safety	3.52
Environmental sustainability	3.78
Financial savings	4.30
Expectations of team members	4.52

^*^Likert score “1” was defined as “most motivating,” and “6” was defined as “least motivating”

## DISCUSSION

Using a multifaceted behavioral and educational intervention emphasizing high-value care and environmental sustainability, this 6-month pilot covering 16 inpatient beds produced statistically significant decreases in the average number of basic lab tests and venipunctures per patient per day. The associated estimated 81-kg reduction in CO_2_ emissions is equivalent to driving the average gasoline-fueled car from Boston to New York City or about 6500 smartphone charges. Hypothetically scaled across a 1000-bed hospital over 1 year, potential annual CO_2_ savings could amount to 10,125 kg CO_2_, enough to drive approximately 25,800 miles or charge 818,000 smartphones.^16^

This brief intervention did not show statistically significant shifts in resident perceptions of institutional culture around high-value care, which is unsurprising as it was limited to only one resident team over 1 month. However, it did demonstrate meaningful improvements in residents’ self-perceptions of ordering behaviors, especially how frequently residents reviewed the clinical indications for patients’ lab orders. Statistically significant increases were not seen for second-order actions such as decreasing the frequency or fully discontinuing lab orders for clinically stable patients. This likely reflects appropriate real-life practices where, based on the clinical situation, resident teams decide during their lab review to not de-escalate lab test orders. Alternatively, the survey identified various factors that could have contributed to resident reluctance to de-escalate labs and thus could be targets for future interventions, such as having formal designated times for teams to review lab orders and increasing transparency regarding the cost of laboratory testing.

To our knowledge, this is one of the first published resident-led projects to combine the concepts of high-value care and environmental sustainability to reduce unnecessary lab test ordering. Several other resident-led studies have successfully decreased unnecessary testing using methods such as educating residents on the proper clinical indications for tests,^10^ emphasizing patient experience,^9^ and encouraging mindful lab ordering.^8^ While a limitation of this study’s design is that we cannot attribute the observed behavior change strictly to the environmental education aspect of the intervention, the fact stands that the intervention did make a difference despite crossover of attendings between the intervention and control teams. It is also notable that on post-surveys about 1 month after viewing the environmental education video, environmental sustainability was the most significant factor in resident decision-making after clinical and patient-centered factors. This ranking is appropriate, as physician decision-making should not be guided foremost by environmental impact.

Of note, patient safety outcomes such as ICU transfers and inpatient mortality rates were considered as possible balancing measures to ensure any changes in lab ordering behavior were not associated with increased patient harm. However, the study would have required a substantially larger patient population to achieve sufficient statistical power to assess these outcomes reliably, i.e., with a sufficiently small non-inferiority margin. Several additional limitations are important to note. First, there was one resident who participated in the intervention team twice and thus could have confounded the primary outcome during their second month on the intervention team. The study results were not adjusted to account for this individual. However, it is unlikely that the lab ordering behavior of one resident accounted for the statistically significant differences in lab orders found between the control and intervention groups over the 6 months of the pilot. In addition, there was no formal auditing done to ensure the intervention team was performing the daily team-based reviews of lab orders. Also, while the reduction in venipunctures per day was statistically significant, we did not conduct a patient survey to assess whether this difference was clinically meaningful to patients.

Also of note, the intervention patient cohort was slightly younger than the control patient cohort. It is possible that the residents felt more comfortable de-escalating basic lab tests on younger patients. However, the patient cohorts were equally medically complex based on the Elixhauser comorbidity index, and in our experience, patient age is rarely a factor in daily decision-making around lab test ordering. Patient age was also adjusted for in the propensity models.

Importantly, survey data was self-reported, and a post-intervention follow-up assessment was not conducted to evaluate if observed behavioral changes persisted or waned beyond residents’ time on the intervention team. Evidence that behavioral changes persisted well beyond the intervention period and in the absence of regular email reminders would give greater significance to the pilot results. Also, this was a relatively high-touch intervention involving only a single general medicine service team. Due to the pilot’s small size, relatively high-touch nature of the intervention, and lack of longer follow-up to assess if behavioral changes persisted, this study may have limitations in its replicability, scalability, and generalizability.

Altogether, these results suggest that clinical indications, patient experience, patient safety, *and* environmental sustainability can all be salient levers to utilize in attempts to reduce unnecessary testing and low-value care. Research suggests that how resident physicians practice medicine in residency greatly influences how they practice as attending physicians.^17,18^ Thus, residency represents an opportune time to inculcate residents with high-value and environmentally sustainable care practices. In a world where the total carbon footprint of US healthcare outstrips that of the UK,^6^ education on both the environmental impact of the healthcare industry and sustainable care practices arguably should be integrated into all high-value care efforts and residency curricula.

## CONCLUSION

This pilot of an educational and behavioral intervention integrating the concepts of high-value care and environmental sustainability meaningfully impacted resident ordering behavior, leading to more frequent reviews of patient lab orders, fewer lab tests and venipunctures, and a smaller carbon footprint. These concepts and methods in lab ordering behavior can be incorporated into hospital-wide quality initiatives that aim to limit both unnecessary laboratory testing and excessive greenhouse gas emissions.

## Supplementary Information

Below is the link to the electronic supplementary material.

ESM 1 (PDF 46.1 KB)

ESM 2 (DOCX 19.3 KB)

## Data Availability

The datasets analyzed during the current study are available from the corresponding author on reasonable request.
