# Supplementary material for: Integrating High-Value Care and Environmental Sustainability to Reduce Unnecessary Laboratory Testing by Residents: An Interventional Pilot
Source: J Gen Intern Med. 2025 Aug 4;41(9):2545–50. doi: 10.1007/s11606-025-09776-0 (PMC13305210; doi:10.1007/s11606-025-09776-0)
Supplement: Supplementary file 1 — (PDF 46.1 KB) [file 11606_2025_9776_MOESM1_ESM.pdf]

ITU-B Post-Survey

This survey is about your opinions and practices around ordering basic laboratory tests at Brigham and Women's Hospital. "Basic laboratory tests" refers to the following tests: Complete Blood Count, Basic Metabolic Panel, Liver Function Test, Magnesium, Phosphorus, and PT-INR.

Answers will be kept completely confidential, and results will only be presented in aggregate. Your decision to complete this survey is completely voluntary and will not be known by residency or hospital leadership.

Basic Information

1) What PGY year are you? ○ 1 ○ 2 ○ 3

2) Other than your current ITU rotation, have you already done an ITU rotation in the 2022-2023 academic year? ○ Yes ○ No

3) What is your residency program?
○ Internal Medicine
○ OB/GYN

Assessing Beliefs

Please indicate how much you agree or disagree with the following statements:

4) The BWH IM residency culture around ordering and maintaining basic laboratory test orders embodies high value healthcare principles, e.g. high clinical value, good patient experience, low cost.

◯ Strongly Agree
◯ Agree
◯ Disagree
◯ Strongly Disagree

5) Within BWH IM rotations, there is a cultural expectation to maintain active basic laboratory orders for patients even if labs are not clinically indicated.

◯ Strongly Agree
◯ Agree
◯ Disagree
◯ Strongly Disagree

6) BWH IM residents do not discontinue or decrease the frequency of clinically unnecessary basic lab tests as often as they should.

◯ Strongly Agree
◯ Agree
◯ Disagree
◯ Strongly Disagree

7) When I discontinue or decrease the frequency of clinically unnecessary basic lab tests, I feel my actions are supported by my team members (attendings, fellow, residents, interns).

◯ Strongly Agree
◯ Agree
◯ Disagree
◯ Strongly Disagree

8) I maintain clinically unnecessary basic laboratory test orders on clinically stable patients.

◯ Strongly Agree
◯ Agree
◯ Disagree
◯ Strongly Disagree

Assessing Practices

On a typical general medicine rotation, how often do you perform the following functions:

9) Review and assess the clinical indications for your patients' basic laboratory test orders?

- ◯ At least once daily
- ◯ Multiple times per week
- ◯ Once weekly
- ◯ Less than once weekly

10) Decrease the frequency of any basic laboratory tests for clinically stable patients?

- ◯ At least once daily
- ◯ Multiple times per week
- ◯ Once weekly
- ◯ Less than once weekly

11) Discontinue any basic laboratory tests for clinically stable patients?

- ◯ At least once daily
- ◯ Multiple times per week
- ◯ Once weekly
- ◯ Less than once weekly

12) Some residents believe reducing the amount of basic laboratory tests in clinically stable hospital patients compromises patient safety while other residents do not.

On a 1-7 scale, how much do you believe that reducing the amount of basic laboratory tests in clinically stable patients compromises patient safety?

1 (It does NOT compromise patient safety) — 4 — 7 (It DOES compromise patient safety)

(Place a mark on the scale above)

Assessing Influential Factors

13) Which of the following factors contribute to you NOT discontinuing or spacing out basic lab tests on clinically stable patients? Select all that apply.

- ☐ Discomfort with diagnostic uncertainty
- ☐ Not wanting to miss changes in patient clinical status
- ☐ Lack of clinical experience
- ☐ Ease of ordering repeating labs in Epic
- ☐ Ease of allowing repeat labs in Epic to continue
- ☐ Insufficient time to review patient lab orders
- ☐ Lack of cost transparency of labs
- ☐ Lack of cost-conscious culture at our institution
- ☐ Concern that the attending or other team members will want the data, and I will not have it
- ☐ Lack of role modeling of cost-conscious care
- ☐ Lack of formal education around high value care
- ☐ Clinical recommendations of consultants

Which factors motivated you the most to discontinue or space out unnecessary basic labs?

Please rank all factors.
1 = most motivating
6 = least motivating

		1	2	3	4	5	6
14)	Patient Safety	○	○	○	○	○	○
15)	Patient Experience	○	○	○	○	○	○
16)	Financial Savings	○	○	○	○	○	○
17)	Environmental Sustainability	○	○	○	○	○	○
18)	Lack of Clinical Indications	○	○	○	○	○	○
19)	Expectations of Team Members	○	○	○	○	○	○
