# Supplementary material for: Integrating High-Value Care and Environmental Sustainability to Reduce Unnecessary Laboratory Testing by Residents: An Interventional Pilot
Source: J Gen Intern Med. 2025 Aug 4;41(9):2545–50. doi: 10.1007/s11606-025-09776-0 (PMC13305210; doi:10.1007/s11606-025-09776-0)
Supplement: Supplementary file 2 — (DOCX 19.3 KB) [file 11606_2025_9776_MOESM2_ESM.docx]

**Supplement 2: Table 1. Responses to Survey Questions Assessing Culture and Lab Ordering Behaviors**

|  |  | **Responses (n = 23)** | | | |  |
| --- | --- | --- | --- | --- | --- | --- |
| **Question** | **Study Period** | **Strongly Agree** | **Agree** | **Disagree** | **Strongly Disagree** | **p-Value** |
| Q1. The BWH IM residency culture around ordering and maintaining basic laboratory test orders embodies high value healthcare principles, e.g. high clinical value, good patient experience, low cost. | Pre-Intervention | 8.7% | 39.1% | 43.5% | 8.7% | 0.277 |
|  | Post-Intervention | 13.0% | 52.2% | 30.4% | 4.3% |  |
| Q2. Within BWH IM rotations, there is a cultural expectation to maintain active basic laboratory orders for patients even if labs are not clinically indicated. | Pre | 21.7% | 52.2% | 26.1% | 0% | 0.553 |
|  | Post | 21.7% | 39.1% | 39.1% | 0% |  |
| Q3. BWH IM residents do not discontinue or decrease the frequency of clinically unnecessary basic lab tests as often as they should. | Pre | 30.4% | 52.2% | 17.4% | 0% | 0.886 |
|  | Post | 34.8% | 39.1% | 26.1% | 0% |  |
| Q4. When I discontinue or decrease the frequency of clinically unnecessary basic lab tests, I feel my actions are supported by my team members (attendings, fellow, residents, interns). | Pre | 34.8% | 39.1% | 26.1% | 0% | 0.410 |
|  | Post | 34.8% | 60.9% | 4.3% | 0% |  |
| Q5. I maintain clinically unnecessary basic laboratory test orders on clinically stable patients. | Pre | 13.0% | 60.9% | 26.1% | 0% | **0.028** |
|  | Post | 4.3% | 34.8% | 56.5% | 4.3% |  |

| **On a typical general medicine rotation, how often do you perform the following functions:** | **Study Period** | **At least once daily** | **Multiple times per week** | **Once weekly** | **Less than once weekly** | **p-Value** |
| --- | --- | --- | --- | --- | --- | --- |
| Q6. Review and assess the clinical indications for your patients' basic laboratory test orders? | Pre-Intervention | 8.7% | 39.1% | 34.8% | 17.4% | **0.028** |
|  | Post-Intervention | 13.0% | 73.9% | 8.7% | 4.3% |  |
| Q7. Decrease the frequency of any basic laboratory tests for clinically stable patients? | Pre | 4.3% | 56.5% | 21.7% | 17.4% | 0.150 |
|  | Post | 8.7% | 73.9% | 13.0% | 4.3% |  |
| Q8. Discontinue any basic laboratory tests for clinically stable patients? | Pre | 4.3% | 39.1% | 21.7% | 34.8% | 0.249 |
|  | Post | 8.7% | 52.2% | 17.4% | 21.7% |  |

**Supplement 2: Table 2. Resident-Perceived Barriers to Lab De-Escalation**

|  | **Responses (n = 23)** | |  |
| --- | --- | --- | --- |
| **Factors** | **Pre-Survey** | **Post-Survey** | **p-Value** |
| Discomfort with diagnostic uncertainty | 8 | 12 | 0.312 |
| Not wanting to miss changes in patient clinical status | 15 | 16 | 0.801 |
| Lack of clinical experience | 7 | 6 | 0.801 |
| Ease of ordering repeating labs in Epic | 8 | 6 | 0.613 |
| Ease of allowing repeat labs in Epic to continue | 11 | 10 | 0.801 |
| Insufficient time to review patient lab orders | 10 | 12 | 0.613 |
| Lack of cost transparency of labs | 9 | 12 | 0.448 |
| Lack of cost-conscious culture at our institution | 9 | 12 | 0.448 |
| Concern that the attending or other team members will want the data, and I will not have it | 12 | 10 | 0.613 |
| Lack of role modeling of cost-conscious care | 5 | 4 | 0.801 |
| Lack of formal education around high value care | 8 | 4 | 0.312 |
| Clinical recommendations of consultants | 6 | 9 | 0.448 |
